# The serotype a-EmaA adhesin of *Aggregatibacter actinomycetemcomitans* does not require O-PS synthesis for collagen binding activity

**DOI:** 10.1099/mic.0.001191

**Published:** 2022-05-12

**Authors:** Gaoyan G. Tang-Siegel, David R. Danforth, Jake Tristano, Teresa Ruiz, Keith P. Mintz

**Affiliations:** ^1^​ Department of Molecular Physiology & Biophysics, University of Vermont, Burlington, VT, USA; ^2^​ Department of Microbiology & Molecular Genetics, University of Vermont, Burlington, VT, USA

**Keywords:** adhesin, glycosylation, O-polysaccharides, Gram-negative bacterium, collagen, periodontal disease, endocarditis

## Abstract

*
Aggregatibacter actinomycetemcomitans *, a causative agent of periodontitis and non-oral diseases, synthesizes a trimeric extracellular matrix protein adhesin A (EmaA) that mediates collagen binding and biofilm formation. EmaA is found as two molecular forms, which correlate with the serotype of the bacterium. The canonical protein (b-EmaA), associated with serotypes b and c, has a monomeric molecular mass of 202 kDa. The collagen binding activity of b-EmaA is dependent on the presence of O-polysaccharide (O-PS), whereas biofilm activity is independent of O-PS synthesis. The EmaA associated with serotype a strains (a-EmaA) has a monomeric molecular mass of 173 kDa and differs in the amino acid sequence of the functional domain of the protein. In this study, a-*emaA* was confirmed to encode a protein that forms antenna-like appendages on the surface of the bacterium, which were found to be important for both collagen binding and biofilm formation. In an O-PS-deficient talose biosynthetic (*tld*) mutant strain, the electrophoretic mobility of the a-EmaA monomers was altered and the amount of membrane-associated EmaA was decreased when compared to the parent strain. The mass of biofilm formed remained unchanged. Interestingly, the collagen binding activity of the mutant strain was similar to the activity associated with the parent strain, which differs from that observed with the canonical b-EmaA isoform. These data suggest that the properties of the a-EmaA isoform are like those of b-EmaA, with the exception that collagen binding activity is independent of the presence or absence of the O-PS.

## Introduction

*Aggregatibacter actinomycetemcomitans* is typically associated with periodontitis, an inflammatory condition of the periodontium resulting from dysbiosis of the bacterial biofilm associated with the gingival pocket [1, 2], which leads to the destruction of periodontal tissue. In addition, *Aggregatibacter* species, together with *Haemophilus* species, *Cardiobacterium hominis *, *Eikenella corrodens* and *Kingella* species, are members of the HACEK group of microorganisms, which are Gram-negative oropharyngeal bacteria associated with the causation of inflammation of the endocardium or infective endocarditis [3].

*A. actinomycetemcomitans* synthesizes and expresses an antennae-like surface adhesin, extracellular matrix protein adhesin A (EmaA), which binds to collagen and mediates binding of the bacterium to the exposed extracellular matrix of damaged heart valves [4–6]. This adhesin is also integral for cell-to-cell interactions during microcolony formation of developing biofilms [7]. EmaA is an orthologue of the previously characterized collagen-binding trimeric autotransporter YadA of *Yersinia enterocolitica* [8–10] and is composed of three 202 kDa monomers. The protein is synthesized as a pro-protein in the cytoplasm before transport to the Sec translocon by the interaction of the 56 aa signal peptide sequence mediated by the molecular chaperone SecB [11, 12]. EmaA is classified as an autotransporter type V_c_ secreted bacterial protein [5] wherein the translocated protein, following cleavage of the signal peptide, is targeted to the inner leaflet of the outer membrane by the carboxy terminal ends of the protein [13–15]. Trimerization occurs and forms a membrane pore, which allows for folding of the protein passenger domains during the transition across the membrane, forming the classical antenna-like structures on the surface of the bacterium [6, 13, 15–17].

The canonical EmaA protein was first characterized as a 202 kDa protein consisting of 1965 amino acids, as determined from the genome of a serotype b *A. actinomycetemcomitans* strain [5]. In addition to the atypical size for a prokaryotic protein, existing data suggest that the EmaA monomers are post-translationally modified in the periplasmic space by the WaaL O-antigen ligase, transferring the sugars required for the biosynthesis of the O-polysaccharide (O-PS) in serotype b strains [18]. The presence of WaaL and the O-PS sugars are required for the stability of the adhesin and to maintain the precise 3D structure of the adhesin necessary for collagen binding activity [18–21], although the residue location(s) and the linkage type of the modifications is unknown at this time. Interestingly, modification of the monomers is not important for the role of EmaA in biofilm formation [7].

**Fig. 1.**
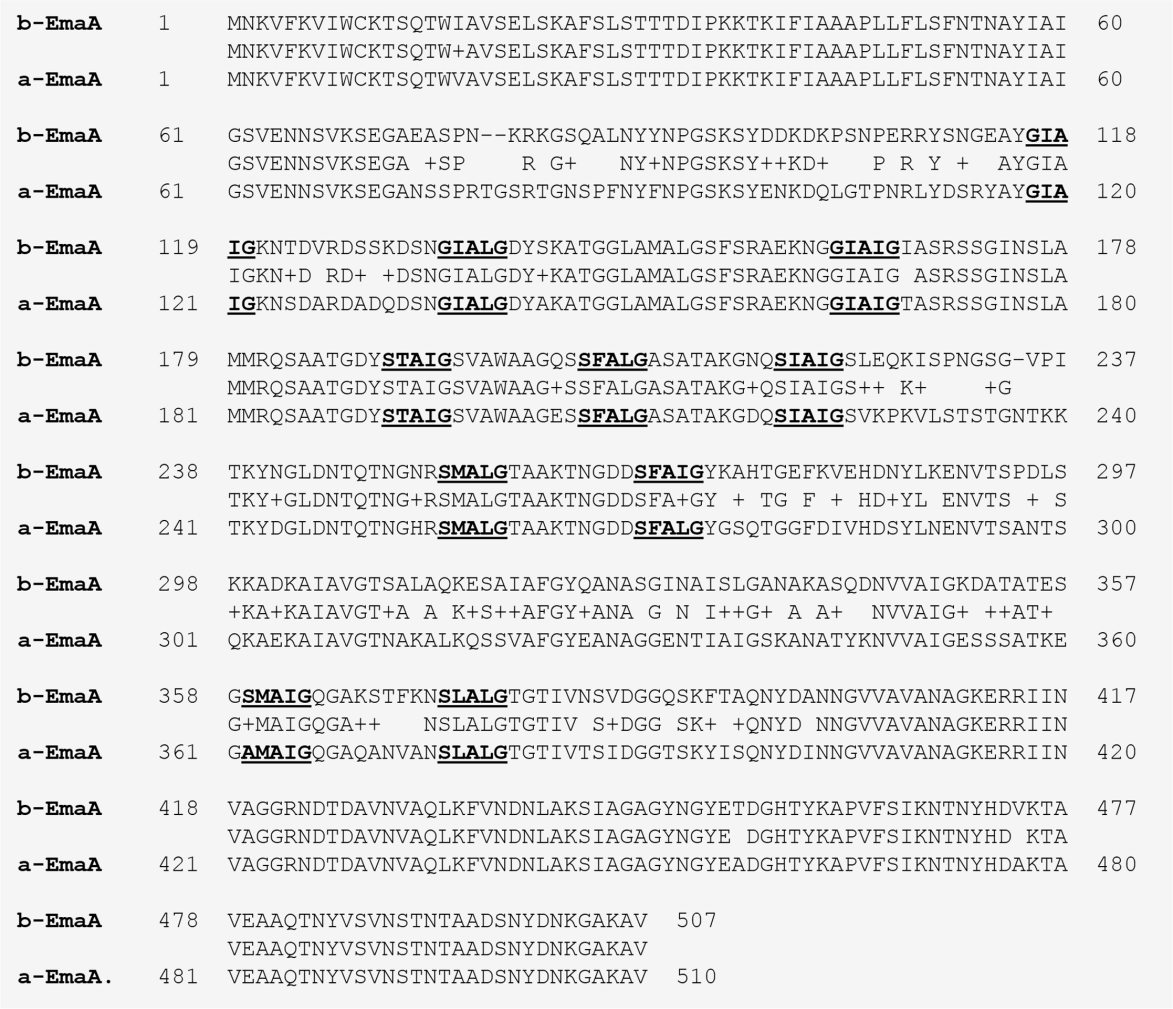
Amino acid sequence comparison of the functional domain of the b- and a-EmaA isoforms. Serotype b: the canonical b-EmaA derived from *A. actinomycetemcomitans* strain VT1169. Serotype a: the a-EmaA isoform derived from *A. actinomycetemcomitans* strain KM708. The first 510 amino acids of each isoform were aligned using the NCBI blast-P suite (BLASTP, RRID:SCR_001010). ‘+’ represents a conservative amino acid substitution. Pentameric repeats crucial for adhesin function are marked in bold and underlined.

A different molecular isoform of EmaA was identified in non-serotype b strains [22]. This shorter, 173 kDa form of the protein is largely associated with serotype a and d strains [22] and is composed of 1679 amino acids. The difference in the mass of the two isoforms is attributed to a deletion of 279 amino acids that correspond to residues 517–795 of the prototypic 202 kDa protein [22]. Furthermore, there is sequence heterogeneity within the N-terminal 427 amino acids, which correspond to the region of the protein associated with the interaction with collagen, when the two sequences are compared (Fig. 1) [22]. The remainder of the protein sequence is highly conserved (>95 % amino acid identity). Deletion of this gene results in loss of collagen binding activity of the bacterium [4–7]. The status of the post-translational modification of the non-serotype b isoform is not known with regard to the structural stability or function of the adhesin.

In this study, we have confirmed that the structures observed on the surface of *A. actinomycetemcomitans* serotype a strains are associated with the presence of *emaA* as visualized by electron microscopy and via gain-of-function studies. Transformation of a serotype a spontaneous *emaA* mutant strain with a plasmid containing a-*emaA* under the control of the endogenous promoter was observed to synthesize EmaA monomers and express antennae-like structures on the surface of the bacterium. A concomitant increase in collagen binding and biofilm formation was observed with the transformed strain when compared to the parent strain. Based on what is known about the post-translational modification status of and the mechanism required for function of the canonical b-EmaA adhesin, studies were conducted to determine if O-PS synthesis is required for full functional activity of the 173 kDa isoform. The serotype a O-PS is composed of disaccharide repeats of d-talose (d-Tal) [23]; therefore, a d-Tal mutant (*tld*) was generated, and the biochemical properties and functional activity of the adhesin were investigated in this mutant background. A distinct decrease in the amount of protein isolated from the bacterial membrane and a change in the molecular mass of the EmaA protein was observed when comparing the *tld* mutant to the parent strain. Additionally, there was no difference in the mass of biofilm formed between the parent and mutant strains. These results are identical to the behaviour of the canonical isoform expressed in serotype b *A. actinomycetemcomitans *. Intriguingly, we observed no difference in collagen binding activity between the *tld* mutant and the parent strain, a result which is contrary to that found with the canonical b-EmaA form of the adhesin. The data suggest that the functional roles of the a-EmaA structures are identical to those of the canonical b-EmaA, and that the modification state of the protein remains important for stability. However, in contrast to the results for the b-EmaA isoform, post-translational modification of a-EmaA is not required for collagen binding activity of this adhesin.

## Methods

### Strains and plasmids

Bacterial strains and plasmids used are listed in Table 1. *A. actinomycetemcomitans* strains were grown statically in 3 % trypticase soy broth, 0.6 % (w/v) yeast extract (TSBYE), with or without 1.5 % (w/v) agar (Becton Dickinson and Company) in a humidified 37 °C incubator with 5–10 % carbon dioxide. All mutants in this study retained growth characteristics similar to the wild-type strain. *Escherichia coli* was grown in broth containing 1 % (w/v) BactoTryptone, 0.5 % (w/v) yeast extract and 1 % (w/v) NaCl (Lysogenic broth, LB; Becton Dickinson and Company) at 37 °C under aerobic conditions with agitation. Targeted genetic mutant strains of *A. actinomycetemcomitans* were maintained at a spectinomycin concentration of 50 µg ml^−1^. Strains containing plasmids were maintained at the following antibiotic concentrations: ampicillin, 100 µg ml^−1^; chloramphenicol, 20 or 1.0 µg ml^−1^ for *E. coli* or *A. actinomycetemcomitans *, respectively; spectinomycin, 50 µg ml^−1^; or kanamycin, 50 µg ml^−1^ .

**Table 1.** Bacterial strain or plasmid details

Name	Description	Reference or source
*** E. coli ***		
DH5αλpir	*endA1 hadR17*(r− m+) *supE44 thi-1 recA gyrA1*(*Nalr*) *relA1* Δ(*lacIZYA*-argF) U169 *deoR* (φ80dlacΔ(*lacZ*)M15) λ pir	[26]
DH10B	F- *mcrA* ∆(*mrr-hsdRMS-mcrBC*) f80*lazZ∆*M15 *DlacX74 recA1 endA1 araD139* ∆(*ara*, *leu*)7697 *galU galK l ^-^ rpsL nupG tonA*	Invitrogen
KM482	DAP auxotroph strain with chromosomal λ pir	A. Goodman, Yale
BL21-DE3	F- *ompT gal dcm lon hsdSB*(*rB-mB-*) λ(DE3 *[lacI lacUV5-*T7*p07 ind1 sam7 nin5*]) [*malB*+]K-12(λS)	Lab strain
*** A. actinomycetemcomitans ***		
VT1281	Non-fimbriated laboratory strain, serotype a	ATCC 29523
KM733	Non-fimbriated strain, serotype b	[7]
KM799	Isogenic *rmlC* mutant of KM733, Spec^r^	[7]
KM354	Non-fimbriated strain, serotype a	[7]
KM802	Isogenic *tld* mutant of KM354, Spec^r^	This study
VT1219	IDH1062: fimbriated clinical isolate, serotype a	M. Saarela, IDH, Finland
KM708	Non-fimbriated variant of VT1219, serotype a	This study
**Plasmids**		
pGEM-T Easy	TA cloning vector, Amp^r^	Promega
pKM02	* E. coli* and *A. actinomycetemcomitans* shuttle vector, Cm^r^	[44]
pKM11	pKM02 containing full-length serotype b *emaA*	[18]
pKM753	pKM02 containing full-length serotype a *emaA*	[7]
pVT1460	Mobilizable plasmid, Kan^r^, requires chromosomal *pir*	[27]
pVT1642	pKM02 containing the VT1169 leukotoxin promoter with a 3′ XhoI site, Cm^r′^	[4]
pKM894	pVT1642 with the *tld* gene ligated into the XhoI site, Cm^r^	This study

ATCC, American Type Culture Collection; Spec, spectinomycin; Amp, ampicillin; Cm, chloramphenicol; Kan, kanamycin.

### Construction of EmaA expression plasmids

Expression of the a-*emaA* was accomplished using a plasmid containing 639 nucleotides of the upstream sequence and the full-length gene (pKM753) [7]. Briefly, a-*emaA* with the putative promoter sequence was PCR-amplified from isopropanol extracted genomic DNA from *A. actinomycetemcomitans* serotype a strain KM708 with the primers CBP1-5′up (5′-ACATGCATGCAACAAATCGCCGTCATCGCC-3′) and 3′EmaAEcoRV (5′-caggatatcGAATAAGCGCATTTTACCA-3′). Immediately after the amplification step, the product was visualized on a 0.6 % agarose gel and the 5.7 kb fragment was gel-extracted (QIAquick Gel Extraction kit; Qiagen) before ligation with pGEM-T Easy (Promega). After growth in *E. coli* DH5α cells, the plasmid was purified (GeneJET Miniprep Kit; ThermoScientific) and the fragment was excised with *Not*I, gel purified and ligated into a shuttle plasmid also digested with *Not*I. Plasmid construction was confirmed by sequencing at the Advanced Genome Technologies Core facility at the University of Vermont. The plasmid used for the expression of b-EmaA (pKM11) was described previously [18].

### Generation of the *tld* mutant strain

The 883 bp *A. actinomycetemcomitans* GDP-6-deoxy-d-talose-4-dehydrogenase (originally identified as GDP-4-keto-6-deoxy-d-mannose reductase, *tld*) [24] gene was PCR-amplified from isopropanol extracted genomic DNA from a non-fimbriated serotype a strain (KM708) using the primers tldfwdXho1 (5′-CTCGAGTCTATGAAAATCTTAGTAAC-3′) and tldrevXho1 (5′-CTCGAGTTAAATCGAAAGCTCCAATAATC-3′) before ligation into pGEM-T Easy and propagation in *E. coli* DH5α cells. The resulting plasmid was isolated and linearized by incubation with *Bgl*II at a unique site contained within the *tld* gene prior to treatment with he Klenow fragment (New England Biolabs). A spectinomycin resistance read through cassette was excised from plasmid pSL60 [25] by incubation with *Sma*I, gel-extracted and ligated into linearized plasmid. After transformation into *E. coli* DH5α and selection on spectinomycin-containing medium, the plasmid was isolated via miniprep and the inactivated *tld* gene construct was released by incubation with *Eco*RI. The DNA was gel-extracted and ligated into the mobilizable pVT1460 plasmid [5]. The resulting plasmid was transformed into diaminopimelic acid (DAP) minus *E. coli* (KM482) [26] which was used as the donor strain for conjugation with *A. actinomycetemcomitans* KM354. Transconjugants were selected on TSBYE-spectinomycin agar plates and verified to be *tld* mutants by colony PCR [5, 25–27].

### Construction of the *tld* gene expression plasmid

The *A. actinomycetemcomitans tld* gene was PCR-amplified and ligated into pGEM-T Easy as described above. The 883 bp sequence was excised via incubation with *Xho*I, gel purified, and the fragment ligated into the same site on the expression vector pVT1642 [4]. The resulting plasmid was confirmed by sequencing. Repeated transformation attempts into the *A. actinomycetemcomitans tld* mutant strain (KM802) were unsuccessful, so the mobilization cassette of pGP704 [27] was amplified by PCR using the primers mob_XbaI_Rev (5′-ataTCTAGAagccgaccaggctttcc-3′) and mob_XbaI_Fwd (5′-agcTCTAGAtttttgtccggtgttgg-3′), purified, incubated with *Xba*I and ligated into the *tld* gene expression plasmid linearized with the same restriction enzyme. This plasmid was transformed into DAP minus *E. coli* for conjugation as described above. Transconjugants were selected on TSBYE medium containing chloramphenicol and confirmed to contain the *tld* gene via PCR.

### Development of anti-serotype a *A. actinomycetemcomitans* antiserum

A polyclonal antibody was developed against a serotype a strain of *A. actinomycetemcomitans* (KM708; Cocalico Biological). Briefly, a pellet of 1.8×10^9^ cells was resuspended in 500 µl ethanol, fixed with 4 % (w/v) paraformaldehyde in PBS (136.9 mM NaCl, 8.1 mM Na_2_HPO_4_, 2.68 mM KCl, 1.46 mM KH_2_PO_4_, 0.46 mM MgCl_2_, pH 7.4) overnight at 4 °C, and rinsed twice using PBS prior to injection into rabbits. After an assessment of multiple test bleeds, the antiserum was collected and immunoglobulins were concentrated by the addition of solid ammonium sulphate to 50 % saturation, followed by centrifugation. The resulting pellet was dissolved in 50 % (v/v) glycerol/water and the protein concentration was determined spectrophotometrically. The concentrated antiserum was stored at −20 °C.

### O-PS characterization of the *tld* mutant strain

BactoELISA assays were performed to characterize O-PS expression [28]. Ten-fold serial dilutions of 10^8^ c.f.u. ml^−1^ bacterial cells grown overnight in TSBYE were added to a 96-well plate (Nunc) and allowed to dry completely. The wells were washed with Tris-buffered saline (TBS; 20 mM Tris-HCl, 150 mM NaCl, pH 7.5), blocked with 0.5 % BSA-TBS, and adhered bacteria were detected using ~3 µg m^l−1^ of the rabbit anti-serotype a *A. actinomycetemcomitans* concentrated antiserum. After 1 h, plates were washed with TBS +0.05 % (v/v) Tween 20 and incubated with the secondary antibody, horseradish peroxidase (HRP)-conjugated goat anti-rabbit immunoglobulin (Jackson Laboratory). Immune complexes were detected by the addition of 100 µl/ per well of peroxidase substrate [4.0 mg *o*-phenylenediamine, 4.0 µl 30 % H_2_O_2_, 10 ml citrate phosphate buffer (0.1 M Na_2_HPO_4_, 0.05 M Na_3_C_6_H_5_O_7_, pH 5.0)] and the reaction was terminated by the addition of 50 µl per well of 4.0 M sulphuric acid. The absorbance was quantified at a wavelength of 490 nm using an ELx800 plate reader (BioTek). Experiments were performed in triplicate with at least three biological replicates; a one-way ANOVA test was used to identify statistical significance (*P*<0.05).

### Fractionation of membrane and cytosolic proteins

The whole membrane protein fraction of *A. actinomycetemcomitans* was prepared as described previously [5, 22]. Briefly, 200 ml late logarithmic-phase cells were harvested, washed with PBS, and resuspended in 2.0 ml of 10 mM HEPES (pH 7.4) with 1.0 mM PMSF (USB) and Pierce protease inhibitor with EDTA (ThermoFisher Scientific). The bacteria were lysed by three cycles of 9000 p.s.i. (62 100 kPa) on ice using a French pressure mini cell. Whole cell lysates were centrifuged at low speed at 10 000 ***g*** for 30 min to remove unbroken cell debris, followed by ultracentrifugation at 100 000 ***g*** for 1 h to separate bacterial insoluble membrane proteins (the pellet) and the soluble cytosolic proteins (the supernatant). The pellet was washed with HEPES, and then ultracentrifuged again to further remove contaminated cytosolic proteins. The same ultracentrifugation was also repeated for the supernatant to remove contaminated membrane proteins. The protein concentration was measured using the Evolution 201 UV-visible spectrophotometer (ThermoFisher Scientific) and measured at an absorbance of 280 nm.

### SDS-PAGE and immunoblotting

Equivalent amounts of protein were prepared in buffer containing a final concentration of 5 % (v/v) beta-mercaptoethanol, 0.06 M Tris pH 6.8, 10 % (v/v) glycerol, 0.02 % (w/v) bromophenol blue, and 2 % (w/v) SDS; boiled for 5 min, and loaded into 4–15 % polyacrylamide Tris-Glycine minigels (Bio-Rad). The separated proteins were transferred to 0.2 µm Immobilon-PSQ PVDF membrane (MilliporeSigma) at 90 V, 4 °C for 90 min, and probed with an anti-EmaA stalk monoclonal antibody [22]. The immune reactive complex was detected using HRP-conjugated goat anti-mouse IgG (Jackson Laboratory), and visualized using SuperSignal West Pico plus chemiluminescent substrate (ThermoFisher Scientific).

### Immuno-dot blot analysis

Quantification of the expressed isoforms of EmaA was performed using a modified version of the protocol described in Yue *et al*. [29]. Briefly, bacterial membranes were isolated as described above. Equal amounts of membrane proteins were immobilized on 0.2 µm pore size nitrocellulose membranes (Amersham Protran) using an immuno-dot blot apparatus (Bio-Rad Laboratories). The membrane was washed with TBS, incubated with 5 % (w/v) non-fat milk for 1 h to block, and finally washed with TBST. Purified monoclonal antibody [22] was applied to the membrane and incubated for 1 h and the membrane was visualized as described above. The dot signal intensities were quantified using ImageJ software.

### Transmission electron microscopy (TEM) using whole-mount negatively-stained bacteria

*A. actinomycetemcomitans* cells were prepared as described previously [6]. Briefly, bacteria were recovered from −80 °C and grown on TSBYE plates for 2–3 days. A single colony from each strain was inoculated into 10 ml of TSBYE broth and grown for 16 h, diluted to 1 : 10 and grown for an extra 150 min. Bacteria were collected by centrifugation of 1.0 ml bacterial suspension at 1000 ***g*** for 1 min at 4 °C, and resuspended in 100 µl PBS pH 7.4. Then, 5.0 µl aliquots of the bacterial suspension were placed onto 300 mesh carbon-coated grids, and the grids were rinsed twice with 6 µl of buffer. The grids were stained using Nano-W staining solution (Nanoprobes) in the following manner: after excess liquid was wicked from the sample, a 6 µl drop of stain was added and immediately wicked away, and this process was repeated a second time. Finally, a third stain drop was added and left on the grids for 60 s before the stain was wicked off completely and the grids were slowly air-dried.

Data were collected using a Tecnai12 electron microscope (FEI) equipped with a LaB6 cathode (Kimball Physics), operated in point-mode [6, 30], and a 2048×2048 pixel CCD camera (pixel size of 14 µm; TVIPS). All images were recorded on the CCD camera at an acceleration voltage of 100 kV and a nominal magnification of 42 000, which corresponds to 0.308 nm pixel size on the specimen scale. Data were collected under low dose exposure conditions (10 e^−^ Å^−2^) as previously described [31].

### Biofilm assay

Biofilm formation was evaluated using 96-well cell culture plates with Nunclon Delta cell culture treatment (Thermo Fisher) as described previously [7]. Briefly, bacteria were recovered from −80 °C and grown on TSBYE plates for 3 days, and a single colony of each strain was inoculated into 10 ml of TSBYE broth and grown overnight, diluted to 1 : 10 and grown for one doubling time (~150 min). The prepared bacteria of each strain were loaded at ~1.0×10^6^ in 200 µl per well, and grown for 24 h. After the growth period, each well was washed with PBS (pH 7.4) in triplicate, stained with 0.1 % (w/v) crystal violet in water for 20 min and washed to remove the extracellular dye, and solubilized using a 2 : 1 solution of water/glacial acetic acid. Quantification was performed using a spectrophotometer measuring the absorbance at 562 nm. Data were analysed using one-way ANOVA and *P*<0.05 was considered significant.

### Collagen binding assay

The collagen binding activity of different strains was evaluated using 96-well ELISA plates, as described previously [32]. Briefly, Type V collagen from human placenta (Sigma Type IX, C3657; Sigma-Aldrich) was pre-solubilized in 0.5 M acetic acid, diluted in carbonate coating buffer to a working concentration of 10 μg ml^−1^ per well, and 100 µl was applied to individual wells prior to incubation overnight at 4 °C. The wells were rinsed with TBS and blocked with TBS containing 0.5 % BSA. Approximately 1.0×10^8^ bacteria per well were added and incubated for 1 h at 37 °C. The plate was washed thoroughly with TBS and incubated with the *A. actinomycetemcomitans* whole cell primary polyclonal antibody at a concentration of ~3 μg ml^−1^ for the anti-serotype a, and ~5 μg ml^−1^ for the anti-serotype b [5]. Immune-complexed, adhered bacteria were detected as described above. Data were analysed using Student’s *t*-test or a one-way ANOVA test and *P*<0.05 was considered significant.

## Results

### Surface antennae-like projections are encoded by *a-emaA*

**Fig. 2.**
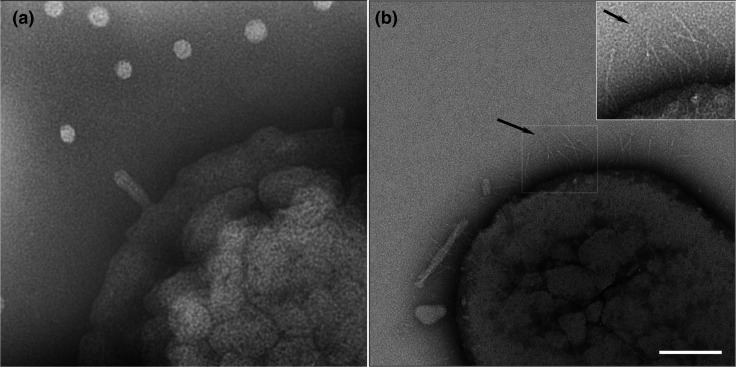
Transmission electron micrographs of whole mount, negatively stained preparations of *A. actinomycetemcomitans *. (**a**) Strain ATCC 29523, serotype a, mutant for EmaA. (**b**) Strain ATCC 29523 transformed with a plasmid expressing a-*emaA* (pKM753). Arrows indicate antenna-like EmaA adhesins. Bar, 100 nm.

ATCC29523 is a serotype a *A. actinomycetemcomitans* strain that contains a spontaneous mutation in *emaA* [22] and does not express antenna-like projections on the surface (Fig. 2a). The isoform of EmaA expressed by serotype a and d strains is encoded by a 5040 nt gene [22]; this sequence was amplified with the upstream 639 bp (the putative promoter) to construct an a-*emaA* expression plasmid. TEM images of strain ATCC 29523 transformed with this plasmid (Fig. 2b) displayed numerous antenna-like surface projections reminiscent of those observed for the serotype b strains expressing canonical b-EmaA [6].

### Collagen binding and biofilm formation activity of the a-EmaA adhesin

**Fig. 3.**
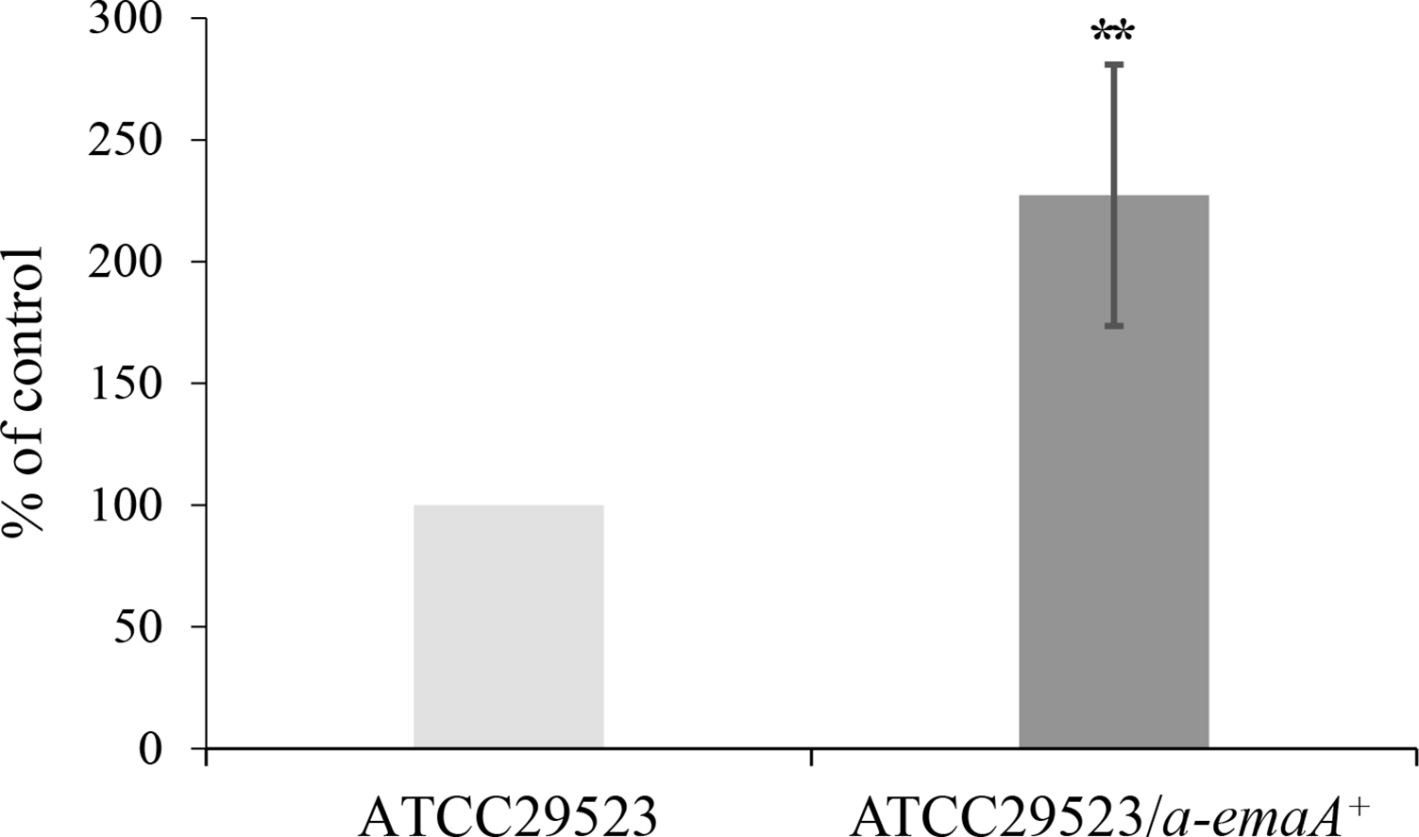
Collagen binding activity of serotype a strains in the presence or absence of the a-*emaA* gene. Light grey: strain ATCC 29523 (mutant for *emaA*); dark grey: strain ATCC 29523 transformed with a plasmid expressing a-*emaA* (pKM753). Collagen binding activity was normalized to strain ATCC 29523 and was set at 100%; a minimum of three biological experiments were performed. Asterisks indicate significance, **P<0.01.

EmaA is associated with collagen binding and biofilm formation [7, 32]. Previously, it was observed that transformation of an a-*emaA* mutant strain with a plasmid expressing a-*emaA* approximately doubled the biofilm mass formed when compared to the original mutant strain (ATCC 29523) [7]. To determine if the a-EmaA isoform manifests with similar collagen binding activity as the b-EmaA isoform, collagen binding assays were performed utilizing the same a-*emaA* mutant and transformed strains. In these experiments, the strain transformed with a-*emaA* demonstrated a >2-fold increase in collagen binding when compared to the mutant strain (Fig. 3).

### Development and characterization of a serotype a O-PS mutant (*tld*) strain

**Fig. 4.**
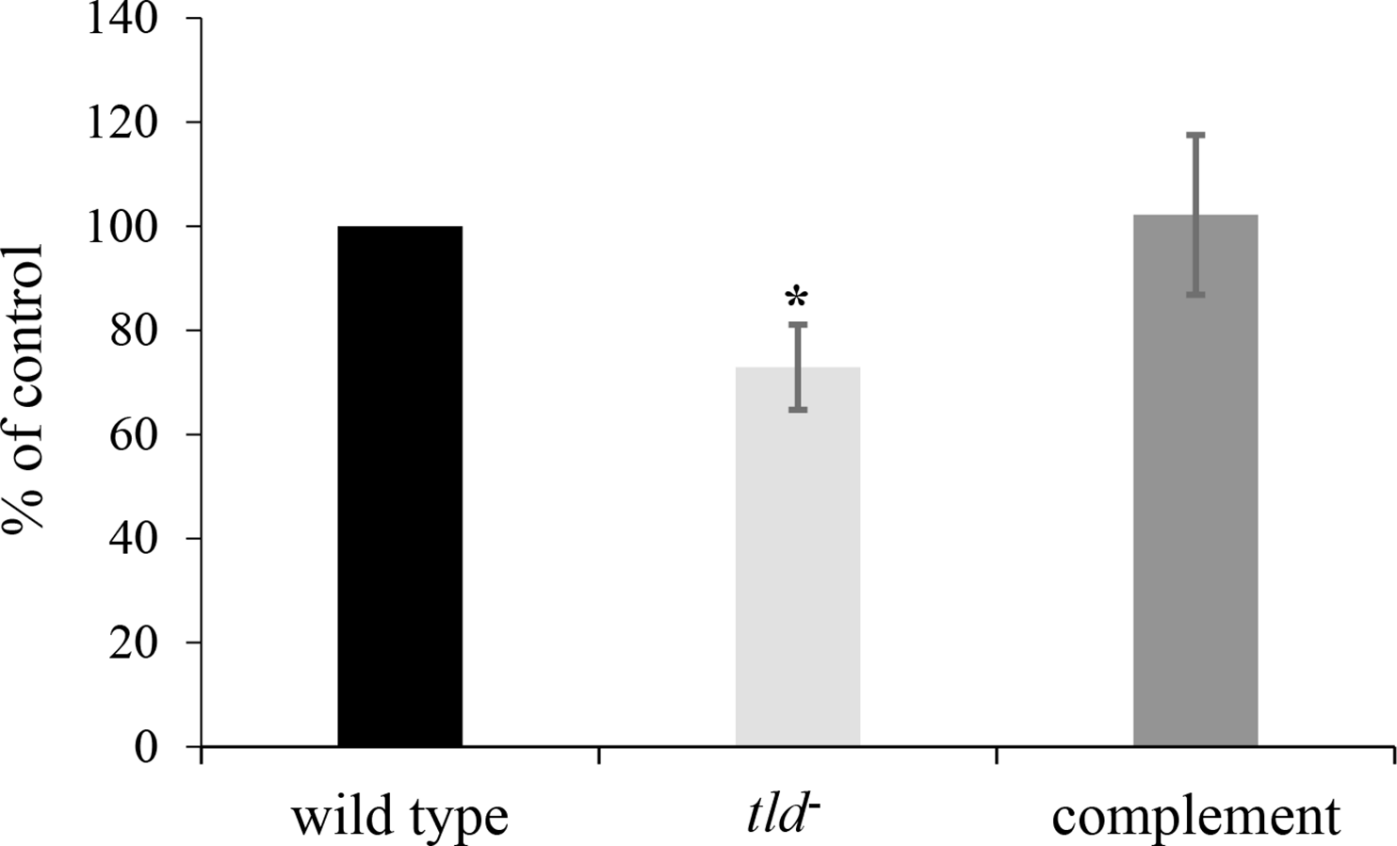
Characterization of O-PS biosynthesis of wild-type, isogenic *tld* mutant and transformed serotype a *A. actinomycetemcomitans* strains. Anti-serotype a concentrated antiserum was used to detect O-PS differences between strains. Wild-type: strain KM354; *tld^-^*: KM354 containing a disrupted GDP-6-deoxy-d-talose-4-dehydrogenase (*tld*) gene; complement: *tld* mutant strain transformed with a plasmid expressing *tld*. Immunoreactivity was normalized to the wild-type and was set at 100 %; a minimum of three biological experiments were performed. Asterisks indicate significance, *P<0.05.

O-PS biosynthesis is required for collagen binding activity and stabilization of the canonical b-EmaA adhesin [18–21]. To determine if the a-EmaA isoform also depends on O-PS biosynthesis, the gene encoding an enzyme essential for the synthesis of the serotype a O-PS, GDP-6-deoxy-d-talose-4-dehydrogenase (*tld*) [33], was inactivated by insertion of an antibiotic cassette encoding spectinomycin resistance. To detect the presence or absence of O-PS, a BactoELISA was used to determine antigenic differences between the parent and *tld* mutant strain. In this assay format, the strain containing the disrupted *tld* demonstrated a 27.1±8.1% reduction in antibody reactivity compared to the parent strain (Fig. 4). Immunological reactivity equivalent to the parent strain was restored following transformation of the mutant strain with a plasmid expressing the wild-type *tld* gene.

### Characterization of EmaA synthesized by the *tld* mutant strain

**Fig. 5.**
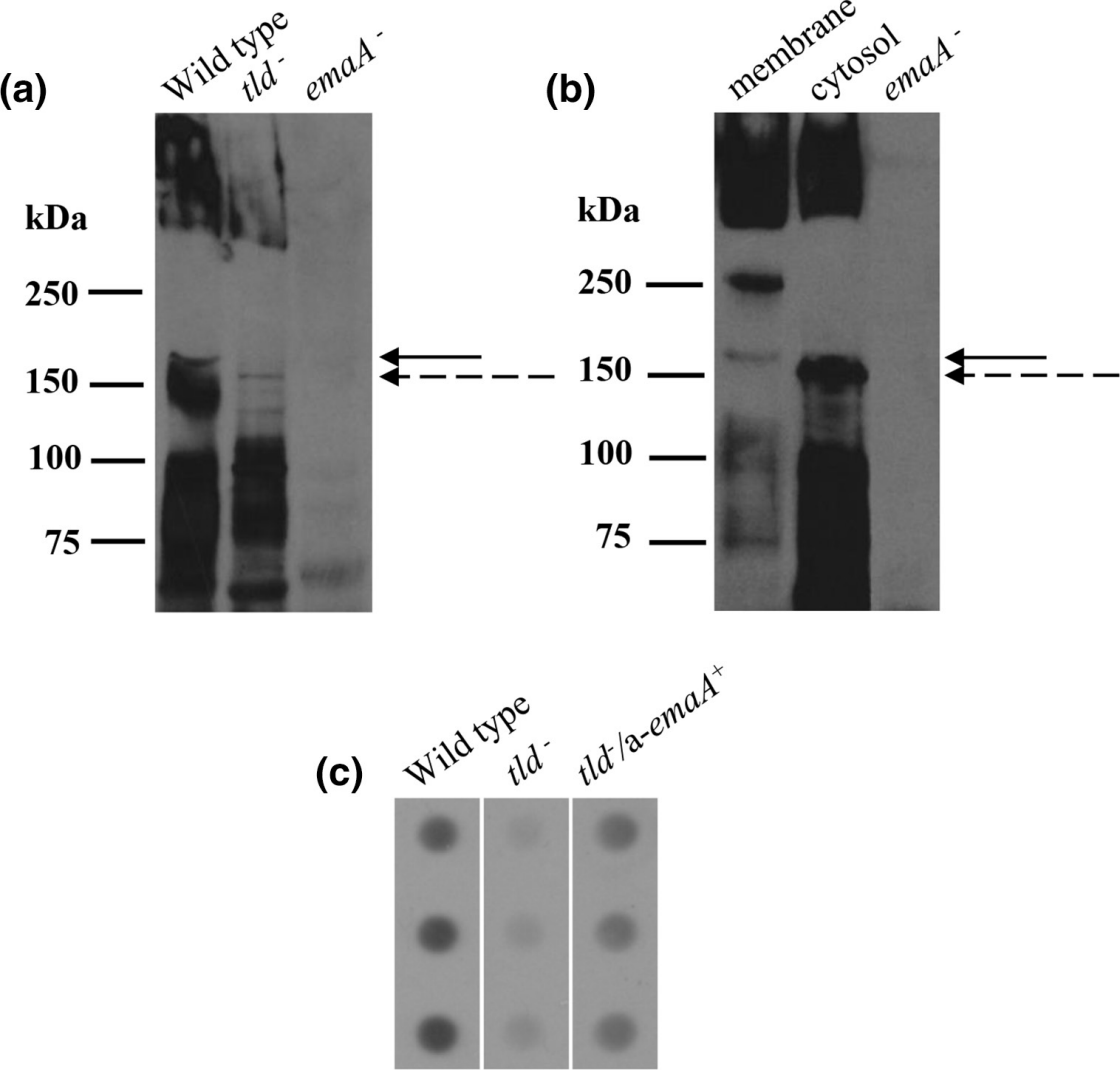
Characterization of EmaA monomers isolated from wild-type and *tld* mutant strains. (**a**) Equivalent amounts of membrane protein from each strain were prepared and separated by electrophoresis using 4–15 % gradient polyacrylamide-SDS Tris-HCl gels. The proteins were transferred to nitrocellulose and probed with a monoclonal antibody specific for EmaA. The solid arrow indicates the electrophoretic mobility of the EmaA monomers associated with the wild-type strain. The dashed arrow corresponds to the mobility of the EmaA monomers associated with the *tld* mutant strain. The immunoreactive material at the top of the immunoblot corresponds to EmaA aggregates associated with the stacking gel. WT: wild type (KM354); *tld^-^*: O-PS mutant strain; *emaA* ^-^: strain ATCC 29523 mutant for *emaA*. (**b**) The membrane and cytosolic fractions were prepared from strain ATCC 29523 expressing *emaA* and analysed as described in (a). The solid arrow indicates membrane-localized EmaA, and the dashed arrow indicates cytosol-localized EmaA. Right-most lane: membrane fraction proteins isolated from strain ATCC 29523 and transformed with an empty plasmid. (**c**) Immuno-dot blot of equivalent amounts of membrane protein from the wild-type strain, O-PS mutant and O-PS mutant strain transformed with plasmid expressing a-*emaA*, probed with the same antibody as in (a).

Changes in the electrophoretic mobility of a protein in SDS-PAGE is a hallmark of the post-translational modification of a protein. Electrophoretic separation of equal concentrations of membrane protein from the parent and *tld* mutant strain followed by immunoblot analysis using an anti-EmaA monoclonal antibody demonstrated differences in the molecular masses and intensity of staining of EmaA. As observed in Fig. 5(a), there is a distinct difference in the electrophoretic mobility of the EmaA monomer (~173 kDa) of the parent strain compared with the *tld* mutant strain. While the majority of the adhesin aggregates and remains located in the stacking gel as protein aggregates [18, 19], the monomeric EmaA can enter the gel. The EmaA monomers from the *tld* mutant strain migrated farther into the gel than those associated with the parent strain. The detected band ladders with lower molecular weight than the monomer were degraded EmaA monomer residues. Furthermore, immuno-dot blot analysis demonstrated that the amount of membrane-associated a-EmaA decreased by ~88 % in the *tld* mutant when compared with the parent strain (Fig. 5c).

The post-translational modification of the canonical b-EmaA protein is suggested to occur in the periplasmic space between the inner and outer membrane [18, 34, 35]. If the a-EmaA isoform is modified in the same way, a difference in the electrophoretic mobility of the cytoplasmic and membrane forms of the a-EmaA would be detected. Immunoblots of proteins isolated from the ATCC 29523/a-*emaA* strain displayed a noticeable difference in the electrophoretic mobility between cytoplasmic EmaA monomers and those associated with the membrane (Fig. 5b). Additionally, immunoreactive materials corresponding to EmaA protein aggregates and incompletely depolymerized oligomer residues were also detected with molecular weights higher than the monomers.

### Biofilm formation and collagen binding activity of the *tld* mutant strain

**Fig. 6.**
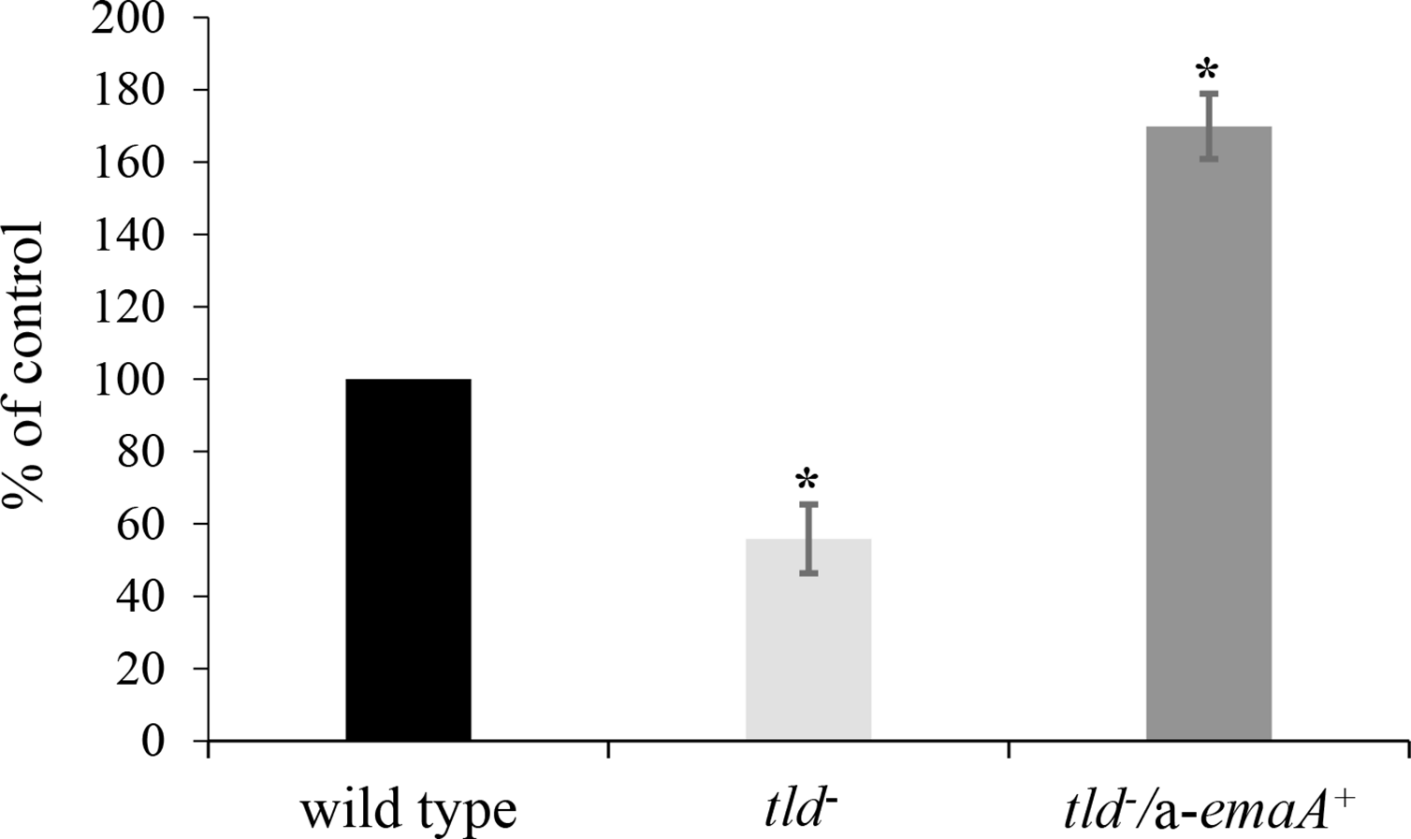
Characterization of biofilm formation in the O-PS-deficient *tld* mutant strain. Wild type: strain KM354; *tld^-^*: the O-PS biosynthesis-deficient mutant; *tld^-^/*a-*emaA^+^*: O-PS mutant strain transformed with a plasmid expressing a-*emaA*. The assay was for 24 h; biofilm formation was normalized to the wild-type strain and was set at 100 %, and a minimum of three biological replicates were performed. Asterisks indicate significance, *P<0.05.

Biofilm and collagen binding were examined in the serotype a O-PS-deficient strain. The *tld* mutant strain demonstrated a 44.2±9.5% decrease in biofilm mass compared with the parent strain (Fig. 6). This decline may be attributed to a reduction in the stability of the adhesin resulting in a decreased amount of EmaA adhesins on the bacterial surface as described above. Therefore, to restore the amount of EmaA expressed on the surface to approximately wild-type levels, the *tld* mutant strain was transformed with a replicating, low-copy-number plasmid expressing the a-*emaA* gene. *In trans* overexpression of *a-emaA* resulted in levels of membrane-associated EmaA that were similar to the parent strain (Fig. 5c), and in this background the mass of the biofilm was restored to that of the parent strain.

**Fig. 7.**
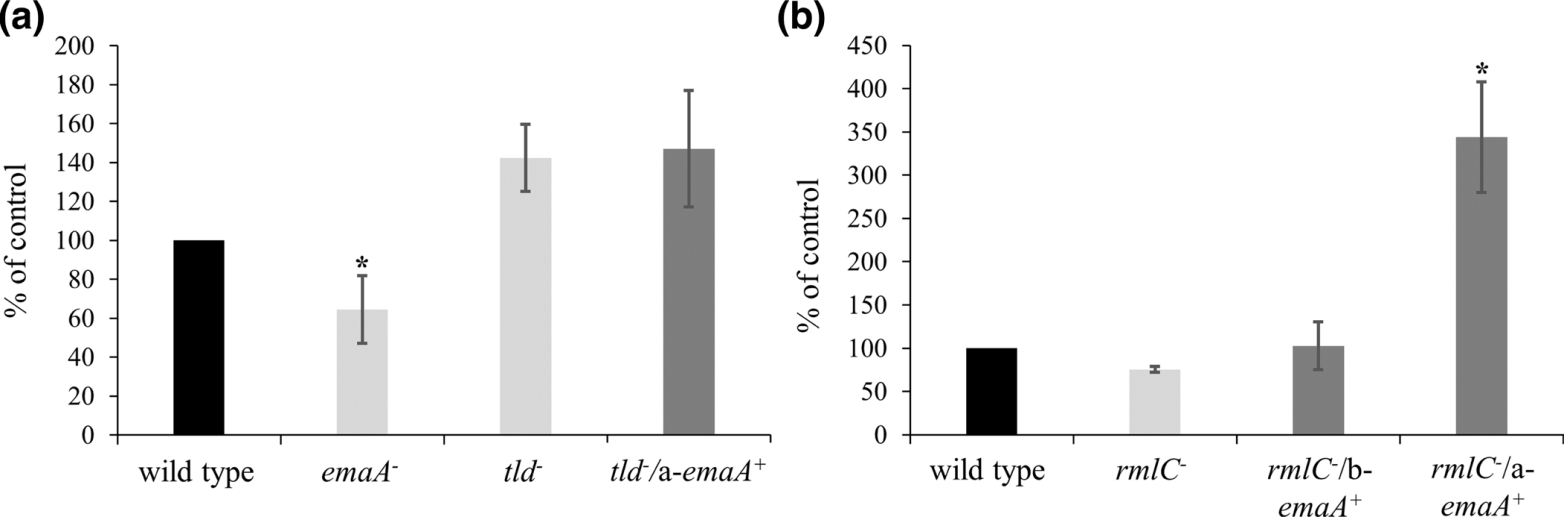
O-PS synthesis and collagen binding activity of *A. actinomycetemcomitans *. Serotype a and b strains deficient for O-PS synthesis were transformed with a plasmid expressing a-*emaA* and were tested for collagen binding activity. (**a**) Serotype a; an anti-serotype b polyclonal antibody was used. Wild-type: strain KM354; *emaA^-^*: isogenic *emaA* mutant; *tld* ^-^: isogenic O-PS mutant strain; *tld^-^/*a-*emaA^+^*: O-PS mutant strain transformed with plasmid expressing a-*emaA*. (**b**) Serotype b; an anti-serotype a polyclonal antibody was used. Wild type: strain KM733; *rmlC^-^*: O-PS mutant strain; *rmlC^-^*/b-*emaA^+^*: O-PS mutant strain transformed with plasmid expressing b-*emaA; rmlC^-^*/a-*emaA^+^*: O-PS mutant strain transformed with plasmid expressing a-*emaA*. Collagen binding activity was normalized to the wild-type strains and was set at 100%; a minimum of three biological replicates were performed for each. Asterisks indicate statistical significance (*P<0.05).

The collagen binding activity of the serotype a *tld* mutant strain was determined via ELISA format using anti-serotype b immunoglobulins to detect cross-reactive surface antigens that are independent of the O-PS composition. Surprisingly, the collagen binding activities of the *tld* mutant and parent strains were observed to be equivalent (Fig. 7a). To compensate for the decreased surface expression of the adhesin in the *tld* mutant, the collagen binding activity of the *tld* mutant/a-*emaA* overexpression strain was also assessed and the strain yielded results similar to the wild-type and the *tld* mutant strains (Fig. 7a). For comparison, the collagen binding activity of an isogenic *emaA* mutant [7] was decreased by 35.5±17.2 % using the same assay.

Previously published data for the canonical b-EmaA isoform closely associated the collagen binding ability of the adhesin to a functional O-PS biosynthetic pathway [6, 18, 19]. To eliminate the possibility that the *tld* mutant collagen binding activity was strain-dependent or an artefact of experimental design, the assay was repeated using a serotype b O-PS-deficient strain with an inactivated rhamnose epimerase gene (*rmlC*) [7, 18, 19] utilizing the anti-serotype a immunoglobulin fraction. In this assay, a 24.5±3.6% decrease in collagen binding ability was observed when the *rmlC* mutant was compared with the serotype b wild-type strain (Fig. 7b). Transformation of the *rmlC* mutant strain with a plasmid expressing b-*emaA* increased the collagen binding activity to levels comparable to that of the parent strain (Fig. 7b). In contrast, transformation of the *rmlC* mutant with a plasmid expressing a-*emaA* via the same promoter and on the same plasmid backbone yielded a 4.6-fold increase in collagen binding activity above the *rmlC* mutant strain and more than double the activity observed for the wild-type strain.

## Discussion

Two isoforms of the EmaA adhesin have been identified and visualized on the surface of multiple *A. actinomycetemcomitans* strains, which appear to segregate according to the serotype of the bacterium [22]. The role of EmaA in bacterial collagen binding and biofilm formation has been well characterized in serotype b strains expressing the canonical 202 kDa monomeric species (b-EmaA) [4–7, 11, 12, 18, 19, 29, 31, 36]. However, there is very little functional information available for the other EmaA isoform (a-EmaA). Comparison of the derived amino acid sequence from the nucleotide sequence suggests that the N-terminal functional domain is similar (~88 % sequence similarity, Fig. 1) to the canonical b-form, with the exception of a 279 amino acid sequence deletion in the carboxyl region within the known functional domain of the b-isoform [22]. The first 60 amino acids of the a-isoform are identical to the b-EmaA sequence and probably contain the signal peptidase cleavage site between A56 and Y57 as previously identified [12]. The remaining sequence shows similarities with several non-conserved amino acid substitutions. However, the hydrophobic pentameric repeats, which are the hallmarks of this class of proteins and are required for the proper folding of the molecules [15, 29, 37], remain highly conserved, whereas variability is present in the intervening sequences. The changes in amino acids may affect the folding of the protein into an active conformation, independent of O-PS synthesis.

As mentioned, all strains of *A. actinomycetemcomitans* contain an *emaA* gene [22]. However, some strains contain amber mutations that result in the synthesis of truncated, prematurely degraded proteins, which are not expressed on the bacterial surface [22]. In this study, we used a serotype a strain (ATCC 29523) that is a mutant for EmaA to confirm the validity of the cloned sequence and the functions associated with this adhesin. Transformation of this strain with the plasmid expressing the full a-*emaA* isoform under the control of the endogenous promoter displayed antenna-like surface projections that are similar to the b-EmaA structures [22]. Consequent to the appearance of these appendages, the transformed strain demonstrated increased collagen binding and biofilm formation compared with the parent strain. The biofilm data are supported by the results reported previously [7]. These phenotypes are identical to what has been observed for the b-EmaA.

However, closer inspection of the electron micrograph images suggests that the a-EmaA structures may differ from the b-isoform [6, 36]. Although the overall structure of the antenna-like appendages is similar, there appears to be subtle differences in the head or functional domain of the two forms. Furthermore, the diameter of the apical end of the a-EmaA appendages appears to be reduced (Fig. 2b), resulting in a thinner domain when compared with the b-EmaA [6, 36]. Additional studies are warranted to support the observations from the 2D images with a complete 3D structural analysis of the functional domain of the adhesin.

The b-EmaA is suggested to be a glycoprotein as determined by various genetic, biochemical and pharmacological approaches [18, 19]. Inactivation of various genes associated with O-PS biosynthesis, including *waaL* [an inner membrane glycosyltransferase which mediates the transfer of the O-antigen from an undecaprenyl-diphosphate (Und-PP) linked intermediate to the core oligosaccharide] [34, 35], an O-PS transport enzyme (*wzt*) [38, 39], and the enzyme associated with rhamnose biosynthesis (*rmlC*) [38, 40], all result in a decrease in the collagen binding activity associated with the b-EmaA isoform [18, 19]. Based on these studies, O-PS sugars (which in serotype b *A. actinomycetemcomitans* consist of branched sequences of d-fucose, l-rhamnose and d-*N*-acetylgalactosamine [41]) are ligated to the protein backbone by the activity of the WaaL ligase.

d-Talose is the sugar associated with serotype a *A. actinomycetemcomitans* O-PS [23, 42]. To investigate the modification of the a-EmaA isoform with serotype a O-PS sugars, a talose biosynthetic mutant was generated by inactivation of the GDP-6-deoxy-d-talose-4-dehydrogenase gene in a non-fimbriated *A. actinomycetemcomitans* strain with a complete chromosomal a-*emaA* gene (KM354). The mechanism for the biosynthesis of d-Tal has been delineated and is derived from α-d-mannose 1-phosphate and GTP in three steps [24, 33], the last of which is the conversion of GDP-4-dehydro-6-deoxy-d-mannose to GDP-6-deoxy-d-talose by the *tld* gene product. The serotype a *A. actinomycetemcomitans *-specific polysaccharide O-antigen is composed of 6-deoxy-d-talose disaccharide repeats, which are acetylated at the *O-*2 position of 1,3-linked 6-deoxy-d-talose [24]; the only other known bacterium to use this extracellular homopolysaccharide is *Pseudomonas plantarii* strain DSM6535 [43]. Interestingly, the stereochemical isomer (6-deoxy-l-talose) comprises the polysaccharide O-antigen of serotype c *A. actinomycetemcomitans *, a serotype which possesses the canonical b-*emaA* isoform [22, 23, 42].

The a-EmaA monomers isolated from the *tld* mutant strain generated in this study demonstrated an increased electrophoretic mobility when compared with the parent strain, a change also observed for b-EmaA in serotype b O-PS-deficient strains [18, 19]. An increase in the mobility of a protein is indicative of a reduction in the mass of the molecule; these changes are usually due to either proteolysis or modifications of the mature protein. In the case of EmaA, the amino terminus has been identified to contain the sequences necessary for the biological activities associated with the adhesin [29], while the carboxyl terminus is necessary to form the pore for translocation across the outer membrane [13–15]. Since the *tld* mutant strain retains the biofilm formation ability characteristic of the EmaA adhesin, it is highly unlikely that the increased mobility of the a-EmaA proteins expressed in that strain are the result of the complete or partial absence of either the amino or carboxyl termini. The possibility that there was a deletion in the intervening sequence is highly unlikely as PCR amplification of the gene yields the expected ~5.7 kbp product (data not shown). The observed difference in the apparent molecular mass of the monomers isolated from the cytoplasm when compared to monomers associated with the membrane (as was observed with the b-EmaA isoform [19]) supports a difference in the mass between these two different physiological locations of the protein. At the same time, the post-translational modification of this trimeric protein leads to a stabilized structure that tends to aggregate with only small numbers of monomers running into the SDS-PAGE separating gel (Fig. 5a), which is also consistent with our earlier observations of the b-EmaA isoform [18, 19].

In addition, we also observed a large decrease in the intensity of the immunological staining of EmaA between the wild-type and *tld* mutant strains. This decrease in staining intensity of the a-EmaA isolated from the *tld* mutant suggests that synthesis of O-PS is necessary for the stability of the protein, a similar phenomenon observed for the b-EmaA isoform in serotype b O-PS-deficient mutants [19]. Together, the data suggest that inactivation of an enzyme (*tld*) associated with the O-PS biosynthetic pathway changes the biophysical properties of the protein and the amount of EmaA associated with the bacterial outer membrane. Therefore, we conclude that the changes we observe in EmaA isolated from the *tld* mutant are due to the lack of any post-translational modifications of the protein that are associated with the presence of the sugars utilized for the bacterial O-PS.

The b-EmaA molecule is required for cell-to-cell interaction in microcolony formation during biofilm development [7] and collagen binding [5, 6]. We have established that the b-EmaA adhesin alone acts to bind the cell to abiotic surfaces and furthermore that this activity is independent of the presence of O-PS [7]. We have found, in this study, that the a-EmaA isoform also acts as an adhesin to abiotic surfaces and is similarly independent of the synthesis of O-PS. The collagen binding activity of the canonical b-EmaA isoform, however, is dependent on O-PS synthesis. Surprisingly, we have confirmed that O-PS synthesis by the cell is not required for a-EmaA collagen binding activity. The collagen binding activity of the *tld* mutant strain was unchanged, even though less EmaA was found associated with the outer membrane, and over-expression of a-*emaA* resulted in no change in the collagen binding activity (Fig. 7a). Furthermore, expression of a-*emaA* in a serotype b O-PS mutant more than doubled the collagen binding activity when compared with the wild-type or the mutant strain expressing b-*emaA*, which is dependent on the biosynthesis of O-PS sugars for activity (Fig. 7b). These data support the hypothesis that O-PS synthesis is not required for EmaA to adopt the optimum conformation necessary for EmaA-associated collagen binding activity in strains expressing the a-isoform.

The absence of O-PS sugar synthesis does not impact collagen binding in strains expressing a-*emaA*, a stark contrast to the necessity of O-PS synthesis for b-EmaA to interact with collagen [19]. The addition of sugars is required to maintain the correct 3D structure of the adhesin, not for direct binding by the sugar moieties [36]. This suggests that the 3D structure of the a-EmaA functional domain is different from the b-EmaA, which may be due to the sequence variation, and is in a ‘locked’ conformation readily amenable for collagen binding. Comparison of the 3D structures of these adhesins would determine the validity of this hypothesis.

The presence of b-EmaA surface structures enhances the ability of the bacterium to bind collagen and form a biofilm, which is crucial for the overall fitness of this microorganism in tissue colonization. The a-EmaA isoform shares considerable attributes with the canonical EmaA associated with serotype b strains. The a-*emaA* codes for a high-molecular-weight protein that forms antenna-like appendages on the surface of the bacterium that are required for collagen binding and biofilm formation. The protein is modified by sugars associated with the O-PS biosynthetic pathway and the modification is required for protein stability. However, the a-EmaA adhesin differs in that O-PS synthesis is not necessary for collagen binding activity. The rationale for the differences in size, sequence and collagen binding mechanisms associated with the a-EmaA compared with the canonical protein is unknown, and the difference(s) between the a- and b-EmaA structures require further studies.
